# Supplementary material for: A Likelihood Approach for Real-Time Calibration of Stochastic Compartmental Epidemic Models
Source: PLoS Comput Biol. 2017 Jan 17;13(1):e1005257. doi: 10.1371/journal.pcbi.1005257 (PMC5240920; doi:10.1371/journal.pcbi.1005257)
Supplement: S1 File — (TAR.GZ) [file pcbi.1005257.s014.tar.gz › HSPH_Online-SI-Revision/PF08/n10-extreme/n10-extreme_PF_table-4.pdf]

1	R0	{2.68692, 12.5884}	{1.86613, 17.0558}	{{1.40482, 11.9489}, {2.32102, 20.}}	{30.5475, 38.4989}	inf	652.	550.436	{270.452, 854.249}	34.2219	0.18013	newly	547.	252.79	{142., 381.}	53.7861	0.365112	Reff	2.38787	1.62813	{1.25801, 2.04702}	31.8166	ar	9089.	7637.65	{5676., 9235.}	16.7863
2	R0	{1.79802, 12.5412}	{1.69632, 18.4668}	{{1.47122, 13.823}, {1.91218, 20.}}	{9.76918, 47.4347}	inf	658.	712.777	{474.385, 950.294}	24.9415	0.101673	newly	408.	286.86	{193., 385.}	30.1373	0.167838	Reff	1.51609	1.39222	{1.23214, 1.52369}	8.78909	ar	7412.	7542.83	{6418., 8436.}	9.39193
3	R0	{2.6174, 10.1105}	{1.9795, 16.7631}	{{1.43491, 10.881}, {2.56822, 20.}}	{25.6948, 67.4338}	inf	487.	449.771	{185.72, 844.922}	42.1738	0.208216	newly	540.	225.07	{82., 358.}	58.4315	0.435269	Reff	2.41377	1.81483	{1.34962, 2.28621}	25.6132	ar	9109.	7923.12	{5902., 9442.}	14.9193
4	R0	{2.27532, 14.3149}	{1.82953, 17.8735}	{{1.51732, 13.2331}, {2.08715, 20.}}	{19.5926, 28.3618}	inf	1121.	979.613	{603.762, 1344.46}	21.0314	0.107892	newly	626.	403.48	{316., 514.}	35.6486	0.198679	Reff	1.73038	1.37734	{1.20099, 1.5228}	20.4022	ar	8478.	8018.3	{6846., 8868.}	7.90399
5	R0	{2.96649, 18.0674}	{2.16478, 17.5004}	{{1.62693, 10.6728}, {2.68759, 20.}}	{27.0254, 14.7357}	inf	1477.	1112.36	{651.759, 1583.56}	28.7754	0.164676	newly	689.	506.31	{339., 661.}	27.2206	0.151487	Reff	2.25186	1.72253	{1.32901, 2.03675}	23.5064	ar	9476.	8943.36	{7464., 9908.}	7.53778
6	R0	{2.94778, 16.0002}	{1.98675, 17.3039}	{{1.43822, 12.7546}, {2.60101, 20.}}	{32.6019, 19.9172}	inf	820.	658.822	{270.921, 1016.15}	32.3864	0.18859	newly	489.	305.02	{137., 456.}	38.7076	0.250182	Reff	2.5348	1.71879	{1.29606, 2.1737}	32.1923	ar	9321.	8042.	{5974., 9486.}	14.3442
7	R0	{2.17562, 11.7184}	{1.95478, 16.7328}	{{1.47983, 11.8516}, {2.46372, 20.}}	{15.9037, 45.6007}	inf	569.	551.641	{274.232, 913.592}	32.7514	0.149781	newly	469.	271.72	{140., 427.}	43.6333	0.283143	Reff	1.95066	1.71928	{1.29321, 2.09348}	15.483	ar	8345.	7990.35	{6091., 9295.}	10.8914
8	R0	{2.7904, 17.8966}	{1.82608, 17.8044}	{{1.48973, 13.4783}, {2.09354, 20.}}	{34.5585, 14.0692}	inf	1183.	697.504	{439.511, 1012.9}	41.7695	0.253399	newly	589.	303.84	{190., 396.}	48.4143	0.303962	Reff	2.25715	1.53886	{1.29875, 1.71975}	31.8229	ar	9285.	8007.31	{6650., 8840.}	13.8362
9	R0	{2.51574, 16.376}	{1.85506, 17.5754}	{{1.4951, 13.0779}, {2.22649, 20.}}	{26.2619, 16.2579}	inf	1086.	847.264	{487.782, 1309.11}	31.3886	0.171332	newly	556.	365.12	{248., 526.}	35.4532	0.207749	Reff	2.01712	1.50634	{1.28062, 1.70646}	25.3221	ar	8937.	8059.97	{6497., 9115.}	10.9138
10	R0	{2.56405, 18.1787}	{1.76214, 17.6832}	{{1.48903, 13.1408}, {2.04913, 20.}}	{31.275, 12.5408}	inf	985.	683.566	{434.665, 969.684}	33.1706	0.188162	newly	476.	294.82	{212., 403.}	38.4832	0.224969	Reff	2.09996	1.48792	{1.30987, 1.6625}	29.1454	ar	9005.	7821.81	{6652., 8763.}	13.2243
11	R0	{2.475, 17.1709}	{1.57818, 19.514}	{{1.49392, 18.7626}, {1.67557, 20.}}	{36.2351, 14.362}	inf	1114.	744.897	{609.34, 884.55}	33.2243	0.179189	newly	539.	280.55	{233., 332.}	47.9499	0.287535	Reff	1.97134	1.26722	{1.23306, 1.32422}	35.7178	ar	8870.	7300.81	{6796., 7736.}	17.691
12	R0	{2.14974, 11.8619}	{1.71598, 18.3419}	{{1.43063, 14.2428}, {2.0639, 20.}}	{20.1773, 54.8649}	inf	514.	543.688	{356.963, 751.917}	23.8864	0.0997567	newly	376.	227.	{147., 317.}	39.6277	0.234444	Reff	1.93176	1.49963	{1.29154, 1.70155}	22.3697	ar	8414.	7537.2	{6224., 8713.}	11.8857
13	R0	{2.20385, 14.4767}	{1.75723, 19.1265}	{{1.54427, 16.5342}, {1.96788, 20.}}	{20.2653, 32.9691}	inf	1178.	1020.37	{713.272, 1354.87}	20.3165	0.102582	newly	681.	388.6	{282., 496.}	42.9369	0.252903	Reff	1.66898	1.29599	{1.18262, 1.38112}	22.348	ar	8347.	8035.29	{7198., 8849.}	6.65856
14	R0	{2.35671, 13.8357}	{2.11636, 17.6343}	{{1.60625, 10.7307}, {2.5859, 20.}}	{13.4774, 33.8322}	inf	1020.	1088.84	{641.46, 1523.89}	25.5221	0.111072	newly	683.	491.05	{332., 635.}	28.593	0.159533	Reff	1.89291	1.67651	{1.37152, 1.92365}	13.2639	ar	8719.	8871.04	{7450., 9674.}	8.47437
15	R0	{1.91368, 10.1082}	{1.79081, 18.1392}	{{1.53563, 13.663}, {2.09618, 20.}}	{10.0924, 79.4508}	inf	746.	880.739	{585.889, 1093.63}	27.3194	0.106827	newly	600.	361.41	{253., 425.}	39.765	0.227962	Reff	1.56616	1.3883	{1.21743, 1.52411}	11.576	ar	7486.	7865.95	{6692., 8694.}	9.61221
16	R0	{2.48727, 14.882}	{1.76864, 17.6888}	{{1.47788, 13.3403}, {2.11729, 20.}}	{28.9705, 23.2499}	inf	818.	557.007	{358.756, 890.721}	36.4481	0.208355	newly	517.	248.05	{138., 377.}	52.0213	0.347192	Reff	2.15174	1.55919	{1.32659, 1.79806}	27.5379	ar	8894.	7781.25	{6598., 8929.}	13.31
17	R0	{2.46795, 13.1915}	{2.0693, 17.1718}	{{1.41802, 11.9343}, {2.62291, 20.}}	{20.0069, 35.9287}	inf	685.	656.745	{286.242, 1121.34}	39.0109	0.190313	newly	499.	311.26	{150., 485.}	39.9639	0.258744	Reff	2.17698	1.80368	{1.30157, 2.23331}	19.1537	ar	8796.	8202.37	{5920., 9615.}	12.4869
18	R0	{2.96603, 16.7543}	{1.98481, 17.6538}	{{1.5164, 12.6821}, {2.35406, 20.}}	{33.082, 15.9406}	inf	1177.	841.36	{434.764, 1268.17}	32.4901	0.192557	newly	657.	369.96	{241., 515.}	43.6895	0.268906	Reff	2.40011	1.64675	{1.32568, 1.9196}	31.3887	ar	9394.	8415.61	{6853., 9419.}	10.8883
19	R0	{2.25216, 9.90465}	{1.83095, 17.0435}	{{1.39508, 11.7109}, {2.53898, 20.}}	{22.2901, 72.6636}	inf	358.	393.524	{208.582, 643.259}	41.3025	0.167094	newly	390.	187.5	{90., 298.}	52.359	0.364338	Reff	2.11343	1.67528	{1.29715, 2.25571}	23.0723	ar	8631.	7485.78	{5659., 9377.}	16.1342
20	R0	{2.70837, 18.1923}	{1.75562, 17.339}	{{1.47865, 11.8592}, {2.06211, 20.}}	{35.1778, 14.594}	inf	1099.	662.118	{427.632, 901.112}	41.6176	0.248696	newly	555.	296.07	{187., 398.}	46.6541	0.291096	Reff	2.21924	1.49078	{1.24721, 1.74432}	32.8245	ar	9183.	7686.68	{6410., 8723.}	16.3853
21	R0	{2.30288, 15.5877}	{1.73278, 19.0712}	{{1.55297, 16.0652}, {1.93965, 20.}}	{24.756, 23.793}	inf	1079.	861.178	{621.366, 1143.12}	23.4372	0.1243	newly	546.	337.61	{242., 434.}	38.1667	0.220516	Reff	1.82273	1.37264	{1.25351, 1.45784}	24.6931	ar	8674.	7927.58	{7133., 8730.}	9.31128
22	R0	{2.82208, 10.7544}	{1.87409, 17.0601}	{{1.39197, 12.0585}, {2.58197, 20.}}	{33.8441, 60.5612}	inf	295.	312.797	{159.218, 474.275}	37.5958	0.157149	newly	339.	155.25	{69., 275.}	56.0855	0.401414	Reff	2.6838	1.7688	{1.33736, 2.37183}	34.2364	ar	9333.	7620.45	{5800., 9419.}	18.9518
23	R0	{1.69923, 10.1366}	{1.78329, 17.7626}	{{1.4829, 13.4427}, {2.09286, 20.}}	{10.5336, 75.2323}	inf	645.	832.163	{529.54, 1169.44}	36.4357	0.127144	newly	502.	352.69	{241., 472.}	31.3127	0.17419	Reff	1.4056	1.44107	{1.24229, 1.6137}	8.00204	ar	7055.	7921.86	{6667., 8886.}	14.5239
24	R0	{2.64944, 19.054}	{1.89944, 18.774}	{{1.66176, 14.4285}, {2.09827, 20.}}	{28.3079, 8.3397}	inf	1801.	1162.29	{872.301, 1561.69}	35.4641	0.199519	newly	721.	455.03	{365., 561.}	36.889	0.205285	Reff	1.75684	1.32508	{1.18614, 1.44309}	24.5762	ar	9147.	8255.84	{7463., 8836.}	9.74265
25	R0	{2.53191, 10.9214}	{1.69827, 18.9293}	{{1.47963, 15.8528}, {2.04477, 20.}}	{32.9252, 73.323}	inf	473.	489.058	{331.673, 692.402}	23.5988	0.10017	newly	441.	198.06	{122., 271.}	55.0884	0.366142	Reff	2.32379	1.51046	{1.35624, 1.70914}	35.0002	ar	9009.	7499.93	{6441., 8603.}	16.7507
26	R0	{1.89155, 6.76944}	{2.07974, 17.2328}	{{1.48538, 12.2313}, {2.55648, 20.}}	{19.8201, 154.567}	inf	395.	633.589	{300.295, 1041.34}	70.4074	0.217695	newly	577.	301.41	{151., 470.}	47.9324	0.318452	Reff	1.71999	1.80956	{1.36362, 2.21994}	15.4323	ar	7348.	8315.63	{6235., 9502.}	19.0222
27	R0	{2.63149, 15.6979}	{1.76263, 17.4222}	{{1.47927, 12.6497}, {2.12331, 20.}}	{33.0176, 19.4632}	inf	797.	544.794	{361.847, 792.278}	38.3366	0.21601	newly	482.	246.17	{156., 385.}	49.695	0.321569	Reff	2.28124	1.55289	{1.31036, 1.83226}	31.9277	ar	9104.	7733.19	{6542., 8889.}	15.3112
28	R0	{2.85141, 12.6992}	{2.05803, 17.055}	{{1.42223, 12.2118}, {2.60782, 20.}}	{28.658, 38.1519}	inf	622.	525.128	{188.977, 896.26}	42.3868	0.237494	newly	530.	257.28	{98., 457.}	52.8226	0.38959	Reff	2.57824	1.84698	{1.33427, 2.41651}	28.5581	ar	9317.	8122.3	{6003., 9602.}	14.3349
29	R0	{2.54244, 12.9085}	{1.71731, 18.557}	{{1.47853, 13.5671}, {2.0415, 20.}}	{32.4542, 44.0942}	inf	820.	622.19	{411.85, 833.125}	26.5344	0.145267	newly	612.	255.06	{170., 332.}	58.3235	0.394539	Reff	2.19184	1.45799	{1.29309, 1.64528}	33.481	ar	9034.	7536.4	{6346., 8658.}	16.5774
30	R0	{2.10982, 11.7636}	{1.76206, 18.2978}	{{1.4917, 13.2909}, {2.04974, 20.}}	{16.7384, 55.8037}	inf	762.	735.124	{463.524, 992.821}	23.754	0.10917	newly	521.	305.77	{189., 397.}	41.8215	0.251137	Reff	1.77373	1.45553	{1.27556, 1.60747}	17.9394	ar	8224.	7768.07	{6550., 8694.}	8.60469
31	R0	{2.76312, 15.5396}	{2.08579, 17.0446}	{{1.54451, 11.7569}, {2.5569, 20.}}	{24.8673, 20.7662}	inf	1053.	832.168	{423.084, 1278.84}	32.1187	0.179324	newly	607.	391.97	{211., 543.}	36.6672	0.22115	Reff	2.27211	1.72936	{1.33489, 2.09278}	23.931	ar	9220.	8392.06	{6753., 9469.}	10.2848
32	R0	{2.21727, 15.5439}	{1.60099, 19.731}	{{1.53606, 18.8537}, {1.66999, 20.}}	{27.7946, 26.9375}	inf	1094.	855.289	{696.524, 1001.69}	22.3222	0.113655	newly	577.	313.81	{249., 376.}	45.6135	0.269717	Reff	1.72703	1.21652	{1.18672, 1.26429}	29.5599	ar	8359.	7585.22	{7130., 7987.}	9.2913
33	R0	{2.72912, 10.8159}	{1.83097, 16.4548}	{{1.39916, 11.905}, {2.30163, 20.}}	{32.9099, 53.8978}	inf	759.	557.832	{256.858, 931.917}	38.3197	0.223818	newly	733.	266.54	{136., 413.}	63.6371	0.477032	Reff	2.39835	1.59877	{1.2599, 1.98799}	33.3387	ar	9196.	7509.39	{5623., 9055.}	18.8172
34	R0	{2.20928, 10.1308}	{2.21123, 16.6141}	{{1.51017, 10.3307}, {3., 20.}}	{18.9491, 66.2546}	inf	601.	719.664	{303.881, 975.121}	40.9222	0.165413	newly	568.	363.46	{203., 531.}	37.6514	0.226731	Reff	1.96317	1.92957	{1.38843, 2.43787}	16.8179	ar	8388.	8599.84	{6422., 9849.}	12.5594
35	R0	{2.10103, 15.2673}	{1.87552, 18.2044}	{{1.53302, 14.1671}, {2.09071, 20.}}	{11.0743, 23.5543}	inf	1074.	905.71	{533.289, 1230.47}	21.6719	0.11522	newly	563.	375.14	{277., 496.}	33.3677	0.187169	Reff	1.64364	1.47545	{1.27031, 1.61915}	10.279	ar	8264.	8183.62	{6843., 8907.}	7.77348
36	R0	{2.74914, 19.2775}	{1.69425, 18.9705}	{{1.49742, 14.1169}, {1.92423, 20.}}	{38.3717, 7.04443}	inf	1325.	802.71	{577.144, 1103.15}	39.7481	0.233594	newly	562.	317.01	{220., 434.}	43.5925	0.261777	Reff	2.13223	1.36664	{1.23405, 1.48914}	35.9057	ar	9301.	7762.53	{6837., 8712.}	16.5646
37	R0	{2.78161, 16.0136}	{1.86423, 18.0331}	{{1.50188, 13.8245}, {2.19278, 20.}}	{32.9803, 18.4659}	inf	1098.	751.898	{432.205, 1080.54}	34.5231	0.200469	newly	605.	323.99	{204., 470.}	46.4479	0.292945	Reff	2.28481	1.55197	{1.33841, 1.73872}	32.0745	ar	9240.	8043.39	{6587., 8985.}	13.0124
38	R0	{2.4454, 8.33501}	{1.80276, 17.24}	{{1.40556, 13.1322}, {2.29589, 20.}}	{27.0592, 106.839}	inf	351.	385.516	{204.355, 616.594}	36.9471	0.148085	newly	491.	180.8	{92., 300.}	63.1772	0.474459	Reff	2.30772	1.64687	{1.30934, 2.11773}	28.8297	ar	8853.	7433.79	{5760., 9056.}	17.3118
39	R0	{2.9154, 12.3861}	{2.03827, 17.5611}	{{1.53452, 13.184}, {2.55721, 20.}}	{30.4344, 42.8582}	inf	746.	573.416	{264.363, 955.499}	38.3256	0.210505	newly	645.	268.09	{136., 468.}	58.4419	0.425658	Reff	2.57605	1.80496	{1.42799, 2.21412}	29.9331	ar	9371.	8211.93	{6429., 9461.}	13.2525
40	R0	{2.40923, 15.1963}	{1.94922, 17.3889}	{{1.52481, 12.0429}, {2.32123, 20.}}	{19.0978, 22.7847}	inf	1012.	973.589	{513.478, 1482.65}	30.0251	0.142336	newly	547.	424.65	{279., 590.}	25.8556	0.141456	Reff	1.91847	1.53327	{1.22517, 1.73533}	20.0786	ar	8735.	8348.79	{6839., 9442.}	9.20675
41	R0	{2.63137, 15.238}	{1.87572, 18.6882}	{{1.58251, 14.2605}, {2.09049, 20.}}	{28.717, 25.9801}	inf	1144.	882.167	{535.009, 1179.17}	25.6857	0.140122	newly	680.	356.6	{253., 459.}	47.5588	0.291585	Reff	2.09194	1.4915	{1.30849, 1.62248}	28.7027	ar	9150.	8193.98	{7034., 8887.}	10.4483
42	R0	{2.48769, 19.4593}	{1.65665, 19.172}	{{1.47721, 14.58}, {1.8307, 20.}}	{33.4061, 5.25519}	inf	1677.	979.569	{709.628, 1169.65}	41.588	0.240935	newly	673.	366.4	{291., 425.}	45.5572	0.269339	Reff	1.67844	1.18534	{1.1163, 1.23726}	29.3788	ar	8932.	7632.54	{6787., 8203.}	14.5484
43	R0	{2.61199, 10.214}	{1.96359, 17.2088}	{{1.40945, 12.5362}, {2.57793, 20.}}	{25.6885, 69.38}	inf	259.	342.129	{168.999, 549.071}	48.3569	0.167373	newly	315.	165.41	{75., 255.}	49.0317	0.332668	Reff	2.50594	1.82274	{1.33798, 2.44577}	27.6224	ar	9061.	7830.84	{5660., 9460.}	15.4998
44	R0	{2.50994, 18.3382}	{1.61147, 19.6969}	{{1.51472, 18.9754}, {1.70565, 20.}}	{35.7967, 7.89227}	inf	1225.	818.72	{625.199, 994.469}	33.2916	0.18199	newly	597.	303.16	{238., 364.}	49.2194	0.301201	Reff	1.9314	1.26977	{1.22936, 1.33647}	34.2567	ar	8827.	7472.26	{6872., 7967.}	15.3477
45	R0	{2.44283, 16.8338}	{1.8197, 17.8579}	{{1.53994, 13.2246}, {2.07279, 20.}}	{25.5086, 16.6401}	inf	1233.	859.979	{526.765, 1199.94}	32.1033	0.182078	newly	606.	367.11	{268., 502.}	39.4208	0.232073	Reff	1.87707	1.4525	{1.27278, 1.62553}	22.6188	ar	8878.	7990.14	{6800., 8864.}	10.3055
46	R0	{2.51815, 18.1462}	{1.7649, 18.829}	{{1.54433, 13.9288}, {2.08964, 20.}}	{29.9129, 11.4954}	inf	1241.	909.241	{596.07, 1189.2}	27.4421	0.152705	newly	572.	358.69	{249., 449.}	37.3374	0.213731	Reff	1.943	1.38028	{1.23912, 1.51197}	28.9618	ar	8978.	8073.39	{7106., 8916.}	10.4575
47	R0	{2.88885, 16.8987}	{1.67359, 18.2042}	{{1.4271, 13.2868}, {1.91589, 20.}}	{42.0672, 16.7369}	inf	890.	568.677	{369.671, 771.172}	36.8403	0.216244	newly	505.	239.01	{157., 323.}	52.6713	0.342666	Reff	2.47083	1.43835	{1.26459, 1.61149}	41.7868	ar	9337.	7372.82	{6089., 8467.}	21.0365
48	R0	{2.58709, 13.8786}	{1.84439, 17.3437}	{{1.49933, 12.58}, {2.30085, 20.}}	{28.7451, 29.7782}	inf	706.	531.19	{296.104, 898.123}	36.0876	0.201471	newly	483.	247.37	{129., 375.}	49.0538	0.328	Reff	2.27612	1.66155	{1.37836, 2.0005}	27.0008	ar	9146.	7955.79	{6604., 9330.}	13.9841
49	R0	{2.42959, 10.7132}	{1.83956, 17.2298}	{{1.40442, 12.8044}, {2.52885, 20.}}	{25.7768, 61.739}	inf	413.	424.328	{220.678, 706.04}	38.3249	0.163272	newly	380.	202.45	{92., 335.}	49.0816	0.333075	Reff	2.25199	1.67543	{1.26438, 2.1582}	26.3994	ar	8863.	7514.89	{5706., 9351.}	17.0828
50	R0	{2.60513, 13.2796}	{1.72402, 17.8995}	{{1.45543, 13.2446}, {2.07198, 20.}}	{33.822, 36.4239}	inf	466.	484.369	{317.729, 683.732}	26.9767	0.110966	newly	376.	210.21	{131., 284.}	44.8005	0.276828	Reff	2.3863	1.5497	{1.31551, 1.82939}	35.0583	ar	9095.	7575.77	{6259., 8808.}	16.8388
